# A Dynamic Network Model to Explain the Development of Excellent Human Performance

**DOI:** 10.3389/fpsyg.2016.00532

**Published:** 2016-04-20

**Authors:** Ruud J. R. Den Hartigh, Marijn W. G. Van Dijk, Henderien W. Steenbeek, Paul L. C. Van Geert

**Affiliations:** Department of Psychology, Faculty of Behavioural and Social Sciences, University of GroningenGroningen, Netherlands

**Keywords:** complexity, simulation models, dynamic systems, expertise, giftedness, idiosyncratic patterns, skewed distributions, talent development

## Abstract

Across different domains, from sports to science, some individuals accomplish excellent levels of performance. For over 150 years, researchers have debated the roles of specific nature and nurture components to develop excellence. In this article, we argue that the key to excellence does not reside in *specific* underlying components, but rather in the *ongoing interactions* among the components. We propose that excellence emerges out of dynamic networks consisting of idiosyncratic mixtures of interacting components such as genetic endowment, motivation, practice, and coaching. Using computer simulations we demonstrate that the dynamic network model accurately predicts typical properties of excellence reported in the literature, such as the idiosyncratic developmental trajectories leading to excellence and the highly skewed distributions of productivity present in virtually any achievement domain. Based on this novel theoretical perspective on excellent human performance, this article concludes by suggesting policy implications and directions for future research.

## Introduction

The topic of excellent human performance is of interest in a wide variety of domains, such as sports, technological creativity, music, arts, and science. Excellence can be conceptualized as domain-specific superior performance, and within the population there are very few individuals reaching exceptional achievements, such as Mozart in the domain of music composition, Einstein in science, and Roger Federer in sports (e.g., Simonton, 1999a, 2000; O'Boyle and Aguinis, 2012). For over 150 years, researchers have attempted to untangle the origins of excellence, in terms of the explanatory components, and the debate on the origins exists ever since (see below). In this article, we propose a novel perspective on excellence and its development, which builds upon the traditions of the dynamic systems approach to social and developmental psychology (e.g., Van Geert, 1991, 1994; Vallacher and Nowak, 1997; Nowak and Vallacher, 1998), in combination with more recent applications of network approaches (e.g., Cramer et al., 2010; Borsboom et al., 2011; Wichers, 2014). We will argue that the explanation for excellent performance does not reside in specific underlying components, but rather in the *ongoing interactions* among the components. Accordingly, excellence development is not “component driven”, but emerges out of idiosyncratic dynamic networks of components (cf. interaction-dominant dynamics; Van Orden et al., 2003). This perspective has consequences for the way in which excellence development should be approached in future research and practice.

### Previous and current attempts to capture excellence

Before elaborating on the dynamic network model, we shall first give a brief overview of the existing literature on excellence, and where necessary, touch upon the related topics of talent and expertise (note that a discussion of the differences between the nature of these concepts is not the aim of this article). Already in 1869, sir Francis Galton published a book on the heredity of genius. Galton (1869) studied the family-trees of eminent scientists, poets, musicians, artists, and athletes, and found that the relatives of these individuals often were excellent performers as well. Therefore, in Galton's (1875) words “there is no escape from the conclusion that nature prevails enormously over nurture” (p. 241). However, around that same time, De Candolle (1873) wrote a book in which he stated that environmental resources (e.g., family, education, facilities), and hence nurture, is the primary explanation for the development of excellence. De Candolle based this conclusion on his observation that excellent scientists, including Galton, were raised under beneficial environmental circumstances, such as high-quality education. He therefore concluded that a stimulating environment (i.e., nurture) is the key to excellence. These early works on excellence gave rise to the famous nature-nurture debate that has featured prominently in the domain of psychology ever since. The identification of specific nature and nurture components underlying excellent human performance has remained a major challenge.

In line with the early ideas of Galton (1869), several researchers hold the view that excellent performance primarily develops out of a specific property in the person, in the sense of some *innate talent* or a *gift* (e.g., Mayer, 2005). This gift is often identified as domain-specific *genetic endowment* (e.g., Bloom, 1982; Winner, 2000; Simonton, 2005). This entails that individuals who have a domain-specific gift (e.g., in science, music, sports, etc.) have the potential to reach excellent performance (e.g., Gagné, 2004, 2013). On the other hand, in accordance with the early view of De Candolle (1873), researchers have postulated that the environment plays a major role. That is, an individual's development of excellent performance can be supported (or limited) by the home environment or the support of a teacher or coach, for instance (e.g., Sloane, 1985; Jussim and Eccles, 1992; Van Yperen, 1995, 1998; Howe et al., 1998; Côté, 1999; Barab and Plucker, 2002; Bloom, 2002; Baker et al., 2003a; Van Bakermans-Kranenburg et al., 2005). A third point of view is that a great amount of effort, i.e., hard work and practice, is required to become an excellent performer. That is, rather than genetic endowment or the environment, many hours of deliberate and high-quality practice are the primary explanation for excellent performance. According to Ericsson and colleagues, becoming an expert often requires more than 10 years of deliberate practice (e.g., Ericsson, 2004; Ericsson et al., 1993; see also Bloom, 1985; Johnson et al., 2006; note that based on a recent meta-analysis, Macnamara et al. (2014) concluded that deliberate practice is not as important as has been argued).

Although the different theoretical propositions have not discarded the role of either genetic endowment or environmental factors and practice, they have—sometimes greatly—differed in the emphasis they put on these factors (see Bloom, 1985; Howe, 1990; Howe et al., 1998; Winner, 2000; Ericsson et al., 2007; Simonton, 2007; Colvin, 2008; Coyle, 2009). However, in general researchers seem to have reached consensus that excellence is multidimensional. In previous years, various models accounting for the multidimensionality from which excellent performance develops have been proposed, such as the Differentiated Model of Giftedness and Talent (DMTG, Gagné, 2004), the Actiotope model (Ziegler and Stoger, 2004; Ziegler, 2005), the Munich Model of Giftedness (Heller, 2007), and Sternberg's WICS (Wisdom, Intelligence, and Creativity, Synthesized) model (Sternberg, 2003). For example, the DMTG stipulates that intrapersonal variables (e.g., physical characteristics, motivation, persistence, adaptability), environmental variables (e.g., social factors, parents, teacher, or coach), and practice all contribute to the development of excellent performance.

In addition to acknowledging the multidimensionality of excellence, some researchers have stipulated that it emerges (and probably changes) across the life span. According to these researchers, excellence should be considered as a property that develops according to dynamic person-environment interactions. This position entails that the emergence of excellence cannot be explained by linear additions of various personal and environmental components, but by (multiplicative) interactions among these components over time (e.g., Walberg et al., 1984; Simonton, 1999a, 2001, 2005; Dickens and Flynn, 2001; Papierno et al., 2005; Lykken, 2006; Davids and Baker, 2007; Phillips et al., 2010). As one example, a high genetic endowment can lead to more successful learning in the domain at issue, leading to active selection of environments that can provide high-quality practice, resulting in even more efficient use of the genetically based learning abilities, and so forth.

Taken together, various components may play a role in the development of excellence. The history of research on the explanatory components underlying excellent human performance, and more specifically the ongoing discussions on the statistical associations between the distributions of particular explanatory components and the distribution of excellence in a particular population, suggests that the search for explanatory components will continue to exist (see O'Boyle and Aguinis, 2012; Kaufman, 2013; Kell et al., 2013; Ericsson, 2014; Hambrick et al., 2014; Macnamara et al., 2014; Plomin et al., 2014; Ackerman, 2014; Simonton, 2014). At the same time, various researchers propose that excellence emerges out of the multiplicative interactions among the components (e.g., Simonton, 1999a, 2001; Phillips et al., 2010). Therefore, in general we argue that a formal model of excellent human performance should consider that multiple components may play a role, but also that multiplicative interactions may take place between the components. The question is, however, how to interpret such multiplicative interactions in the case of excellence development, that is, what is the time-dependent form of these interactions that ultimately result in excellent performance?

Regardless of the specific contributions of particular components, which have been discussed extensively (see above), we argue that a model of excellent human performance should first and foremost fit with properties that are universal across the literature on domain-specific excellence. Indeed, in general, the value of a theoretical model lies in its ability to explain empirical observations that are observed across a wide variety of contexts and situations (Pierce and Aguinis, 2013). In the next section, we will therefore first elaborate on the characteristic properties of excellence. Subsequently, in the following sections we will propose a dynamic, generic model that is a plausible candidate to explain the development of excellent human performance, given these general characteristic properties. Briefly, the model we shall propose is a dynamic network model of ability growth. This means that the ability to develop excellent sport performance, musical performance, scientific performance, and so forth, is embedded in a network of mutually interacting dynamic variables.

We will test the plausibility of the model in light of two classes of properties that are inherent to the topic of excellence (see next section): Its idiosyncratic development over time (i.e., from beginner's level to end-level) and the distribution of excellent performance across domain-specific populations (e.g., sports, music, science). This means that, in this article, we make a distinction between the intra-individual development of excellence—performance potential in terms of the individual's ability level—and the inter-individual differences with regard to the actual performance in terms of specific performance operationalizations. We will demonstrate that simulations of the dynamic network model accurately predict the characteristic properties of excellence. Based on these results, we will discuss the implications of the new conceptualization of excellent human performance—emerging out of idiosyncratic network structures—for future research and practice.

### Developmental properties of excellence across domains

The development of excellence spans a time frame beginning when a domain-specific ability starts to grow (i.e., beginner level) up to the point that superior performance is (repeatedly) demonstrated (e.g., Howe et al., 1998; Simonton, 2001; Abbott and Collins, 2004; Phillips et al., 2010). The growth of abilities ultimately leading to excellence is characterized by a number of qualitative properties, which can be summarized based on Simonton's (2001) review on talent development. First, early indicators of ultimate exceptional abilities are rare to inexistent, and if there are any, they are not particularly reliable. Evidence for this lack of early indicators is found in the fields of sports (Abbott et al., 2005; Davids and Baker, 2007; Vaeyens et al., 2008; Gulbin et al., 2013), music (Sosniak, 1985; Howe et al., 1998; McPherson and Williamon, 2006), arts (Sloane and Sosniak, 1985), and mathematics (Gustin, 1985). However, as performers grow or develop within their particular domain of performance, later excellence becomes an increasingly predictable feature. For instance, within the field of education, tests scores of 12–14 year old students on mathematical abilities, verbal abilities, reasoning, and spatial abilities were found to have predictive value for later education-vocational achievements across a large group of children (Shea et al., 2001).

A second property is that in different individuals a similar ability level may emerge at different ages, and a related third property is that the underlying constituents of a particular ability can change during the person's life span. Fourth, the level of domain-specific ability is not necessarily monotonically rising or stable: It can change or even disappear during a person's life span (see Simonton, 2001). In accordance with the latter three properties, research suggests that individuals may have diverse ways to achieve similar performance levels, thereby emphasizing the idiosyncratic nature of the pathways to excellence (e.g., Simonton, 2000; Abbott et al., 2005; Davids and Baker, 2007; Dai and Renzulli, 2008; Vaeyens et al., 2008; Phillips et al., 2010; Elferink-Gemser et al., 2011; Gulbin et al., 2013). For instance, a Dutch longitudinal project followed the development of (young) soccer players, field hockey players, basketball players, artistic gymnasts, tennis players, and speed skaters (see Elferink-Gemser et al., 2011). In this project, the researchers primarily focused on differences between groups of successful (professional) and less-successful athletes (those children who did not reach the professional status) in terms of average psychological, physiological, technical, and tactical characteristics, at different ages. However, the authors recently argued that the developmental patterns of the athletes involved in the research project were quite idiosyncratic, that is, each athlete seemed to have his or her own unique pathway (Elferink-Gemser et al., 2011; see also the study of Simonton, 2000 on classical music and the study of Gaschler et al., 2014 on performance development in chess).

### Excellent performance across domain-specific populations

At the level of the population (i.e., inter-individual differences in performance), earlier literature has pointed out that one of the most characteristic properties of excellence is its highly skewed distribution (e.g., Simonton, 1999a, 2003). However, because validated tests to determine domain-specific (excellent) abilities hardly exist, we are confronted with the question of how to quantify excellence. To begin with we propose the following general definition of ability: A domain-specific ability is a recurrent and specific pattern of coordination and deployment of skills, psychological components, social components, etc., that are required to demonstrate a particular form and level of performance. This entails that, first, a domain-specific ability does not exist in isolation of other (potentially supporting) components (e.g., Gould et al., 2002; Fischer and Bidell, 2006; Cotton et al., 2011; Van Der Steen et al., 2012). Furthermore, an ability is a latent variable, the nature of which can be defined by the nature of the performance variable that serves as its expression. The measurable level of performance is thus a function of the level of the ability. Therefore, in this article we proceed from the argument that excellent abilities are domain-specific, and that they are manifested in, and measured by, performance accomplishments (e.g., Simonton, 2003; O'Boyle and Aguinis, 2012; Aguinis and O'Boyle, 2014).

In various domains (e.g., arts, science, sports, technology, music, etc.), performance accomplishments can be operationalized by individuals' productivity as defined by *consensual assessment* (e.g., Amabile, 1982; Huber, 2000). Consensual assessment implies that well-specified forms of human performance can be judged by independent domain experts (e.g., reviewers of a research article, coaches of sport teams) and/or based on countable expressions of particular excellent abilities, such as produced scientific articles, musical compositions, or goals made during soccer matches (e.g., Simonton, 1984, 1997, 1999a, 2003, 2014; Walberg et al., 1984; Huber, 2000; O'Boyle and Aguinis, 2012; Aguinis and O'Boyle, 2014). Hence, we used the domain-specific products generated by a particular individual and judged by domain-specific consensual assessment, as an objective—but intrinsically stochastic—and defining indicator of a particular level of excellence in an achievement domain. In line with one of the characteristic properties of excellence, in the next sub-sections we will demonstrate that product distributions in populations of performers are highly skewed to the right (and not bell-shaped) across many achievement domains.

#### Product distributions in soccer

We will start with two illustrative examples of product distribution in one particular domain, namely soccer. In soccer, an incontestable (yet stochastic) indicator of excellence on which data are collected, is a player's total *number of goals* made in between-country matches. Given that this indicator provides a good reflection of excellence only for players whose task it is to attack, and not for players whose task it is to defend, such as a goalkeeper, we also retrieved data on another indicator, namely the *number of inter-country matches* in which players have played. This latter criterion is based on player selection of the coach of the national team and his/her staff, which fits with the definition of consensual assessment (Amabile, 1982). Hence, based on archival data, we will first analyze the product distributions of the two indicators of excellent soccer performance in the population of (former and current) Dutch internationals.

**Figure 1:**
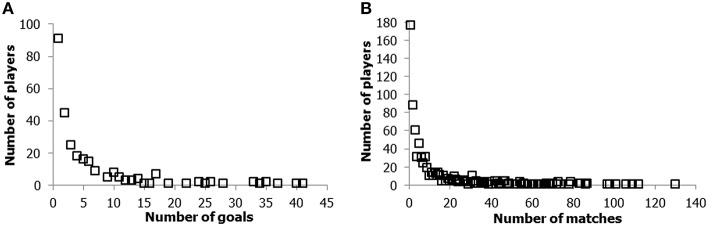
**Distributions of the number of goals made during international matches of the Dutch soccer team against the number of players scoring the corresponding number of goals (A), and the number of international matches a soccer player played in the Dutch national team (B)**.

Figure 1A displays the distribution of Dutch players who have scored during an inter-country match (accessed on March, 2014, from http://voetbalstats.nl/topscnedxi.php). Of the 272 players who scored, 91 scored one goal, 45 scored two goals, and the best two players scored 40 and 41 goals, respectively. The number of goals scored is thus anything but symmetrically distributed (the average number of goals is 5.53). Note that the tail has even expanded toward 50 goals between March 2014 and October 2015, because the Dutch top scorer was still active. Furthermore, as illustrated in Figure 1B, the total number of international soccer matches played by Dutch internationals displays a comparable highly skewed distribution (accessed at March, 2014, from http://voetbalstats.nl/caps.php). A characteristic feature of such asymmetrical and highly skewed distributions is that a representation of the logarithm of the X- and Y-axes (a so-called log-log plot) often approaches a straight line. For this reason, these highly skewed distributions can often be fitted by means of a simple power law equation, which was already discovered in the 1920s as a description of the frequency of the number of scientists who published a particular number of articles (Lotka, 1926). The power law model for the relationship between the number of goals scored or matches played, and the corresponding number of players, is expressed by the following equation:

(1) $$\begin{array}{l} f\left(\text{n}\right)=\frac{\text{c}}{{\text{n}}^{\text{p}}}, \end{array}$$

for *f* (n) the number of players who made n goals or played n matches, c the number of players who made one goal or played one match, n the number of goals or matches, and p a power parameter. Based on the data of the number of goals, c can be set at 91, and an estimation of the p parameter reveals a value of 1.18. Hence, if all frequencies are represented as proportions of the number of players who made one goal (91), the distribution model amounts to the simple function:

(2) $$\begin{array}{l} f\left(\text{n}\right)\approx \frac{91}{{\text{n}}^{1.18}} \end{array}$$

However, various authors have claimed that the power law model does not always provide the best possible fit with the skewed data distributions, and exponential and stretched exponential equations have been suggested as alternative mathematical descriptions of some of the data. These equations produce curved log-log plots instead of the linear log-log plots of the power law equation. The stretched exponential equation states that the probability *p*(x) of observing x products is equal to

(3) $$\begin{array}{l} p\left(\text{x}\right)=Ae^{-{\text{x}}^{\beta }}+B \end{array}$$

**Figure 2:**
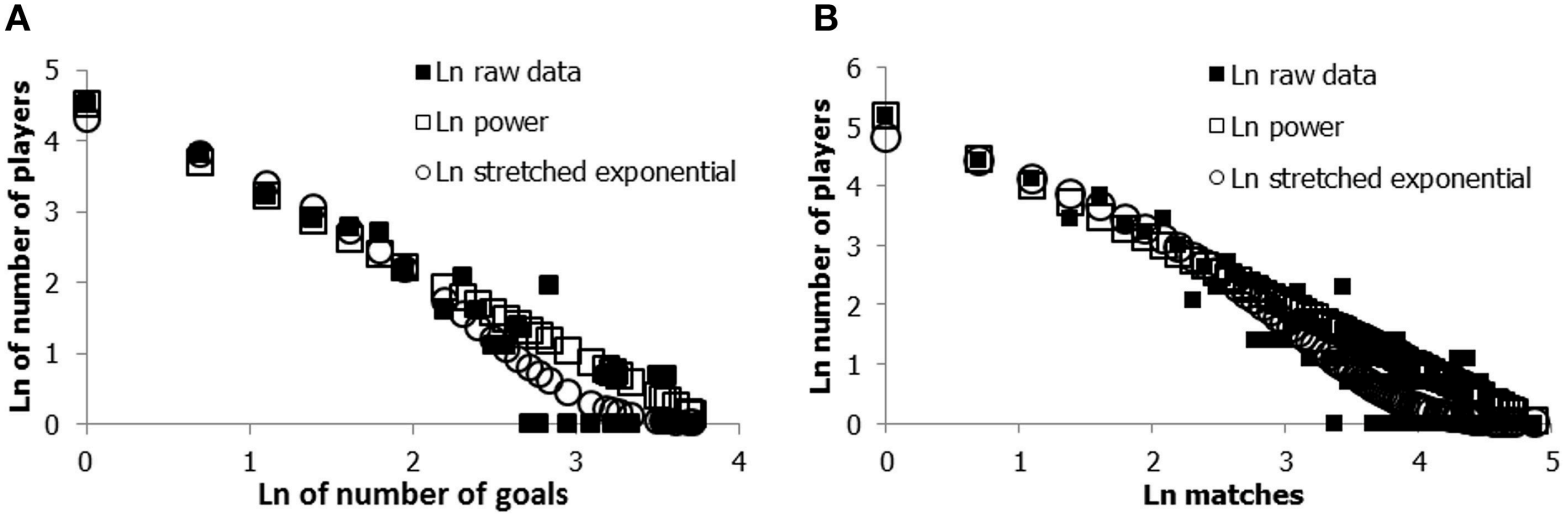
**Log-log representation of the frequency and number of goals (A), and matches played in international matches for the Dutch national soccer team (B), with fitted power law and the stretched exponential models**. Number of goals: Power parameter *p* = 1.18; stretched exponential β = 0.6; Number of matches: *p* = 1.05; β = 0.49.

In both soccer data sets, the power- and the stretched exponential equation provide particularly good fits to the data (see Figure 2). Given that the stretched exponential contains more parameters, it can also fit distributions that deviate from the linear log-log distribution characteristic of the power law model. However, regardless of the specific differences between the two equations, they both signal a characteristic feature of the distribution of performance products, namely that the x-axis (the number of products, in this case the number of goals or matches) spans various orders of magnitude on the natural logarithmic scale. More specifically, with regard to the number of goals the great majority of the (excellent) soccer players who ever scored made only one goal, but a few players—the exceptional cases—have made 30–41 times more goals (in fact, 50 at the moment we submitted this manuscript).

Taken together, the products of excellent soccer performance are highly asymmetrically distributed, according to power laws and stretched exponential distributions. This entails that, among the soccer players, the truly exceptional performers are in the extreme right tail of the asymmetric distribution. Although these illustrations come from one specific sport (soccer), similar distributions apply to sports like American football, cricket, baseball, and basketball (Huber, 2000; Petersen et al., 2008, 2011; De Vany, 2011; O'Boyle and Aguinis, 2012). To demonstrate the universality of these skewed product distributions with regard to excellent human performance in general, we shall also briefly discuss this property in other domains.

#### Product distributions across domains

**Figure 3:**
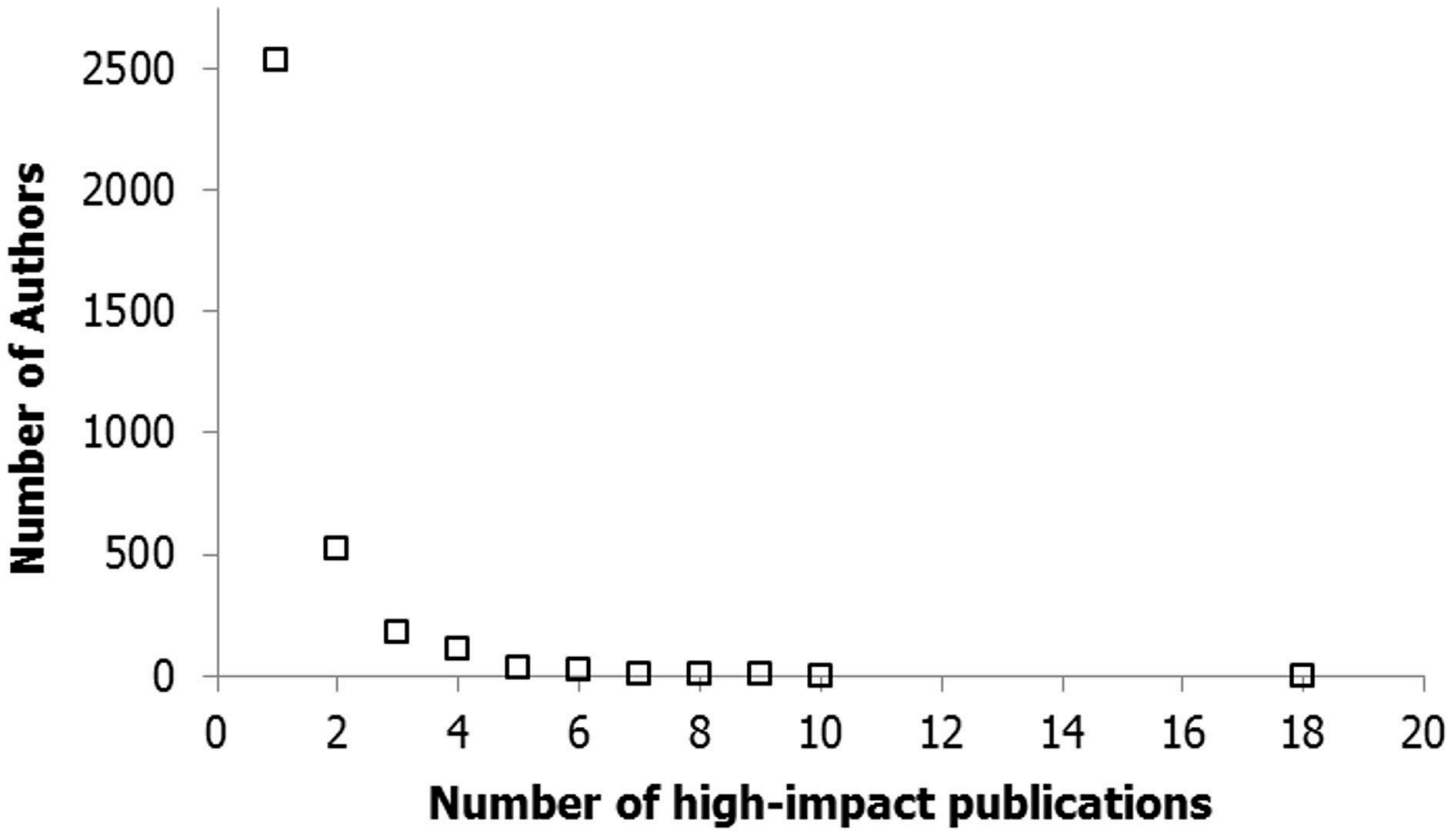
**Product distributions of the number of scientific articles in leading journals in economics vs. the number of authors who published that number of articles, after Sutter and Kocher (2001)**.

In the domain of science, when measuring scientific productivity as the number of papers produced by a particular author, highly right-skewed distributions are revealed in virtually any scientific discipline (Petersen et al., 2010; see Figure 3 for an example from economics). Researchers have discovered power law functions in publication distributions in physics (e.g., Huber and Wagner-Dobler, 2001; Gupta et al., 2005; Nazim and Ahmad, 2008), population genetics (Gupta and Karisiddappa, 1997); mathematical logic (Huber and Wagner-Dobler, 2001), economics (e.g., Cox and Chung, 1991; Sutter and Kocher, 2001; Bino et al., 2002), finance and insurance sciences (e.g., Chung and Cox, 1990; Chung et al., 1992), management science (MacDonald and Kam, 2011), library and information science (Askew, 2008), and psychology (Simonton, 2002; Baker et al., 2003b). Most of the data sets were fitted by a power law distribution with powers ranging from about 1 to more than 3.

Another domain in which performance products show highly skewed distributions, is the number of patented inventions made by individual inventors (e.g., Huber, 2000). In addition, products of musical performance show a similar heavily skewed distribution that can be fitted by power law or stretched exponential equations. More specifically, data on classical composers have been provided by, among others, Huber (2000) and Simonton (1991). Furthermore, O'Boyle and Aguinis (2012) provided evidence for highly skewed distributions of success criteria such as awards and nominations in 17,750 entertainers. The highly skewed distribution has also been found in the field of literature, in which the power law was demonstrated by means of the number of individual titles of monographs, for instance (see Munch-Petersen, 1981). Lastly, O'Boyle and Aguinis (2012) studied the performance of 42,745 candidate politicians in democratic countries, which they defined as a particular politician's election to office. The data of these authors confirm a power law distribution of the performance frequencies.

In addition to the ubiquitous finding that the *output of products* is highly right-skewed in various domains, researchers have shown that the *impact of products* often reveals comparable—sometimes even more extreme—distributions. In the domain of science, for instance, research has shown that the distributions of citations and Hirsch indices of authors (Hirsch, 2005) can be fitted by power law and stretched exponential distributions (e.g., Laherrere and Sornette, 1998; Redner, 1998, 2005; Davies, 2002; Petersen et al., 2010; Spearman et al., 2010; Egghe and Rousseau, 2011; Quigley et al., 2012). Similar distributions were demonstrated in the domain of popular music, in which the researchers examined the (social) impact criterion of number of weeks a record of a particular band remained in the UK top 40 charts (Hamlen, 1991, 1994; Cox and Felton, 1995; Davies, 2002; Fox and Kochanowski, 2004; Spierdijk and Voorneveld, 2009). Finally, literary fame has been found to show extremely right-skewed distributions, using the number of books about poets as an indicator (Martindale, 1995). Although the impact could be considered as an interesting measure of the excellence of the work of a particular scientist, artist, inventor, etc., impact-measures are more of a “sociological” nature. In the current article we decided to focus on individual ability-development, and how abilities lead to the *generation* (i.e., output) *of products*.

#### Conclusion excellent performance across domain-specific populations

In summary, the empirical data collected from a wide variety of fields, including science, sports, technological creativity, literature, classical and popular music, the arts and politics, show that if excellence is operationalized in countable productivity, the distribution is strongly right-skewed, spanning various orders of magnitude. Superior curve fitting is achieved by power law equations and stretched exponential equations. Note, however, that although it is a ubiquitous phenomenon with regard to excellence across domain-specific populations, finding a particular distribution of performance productivity does not entail information about the underlying reasons leading to such distributions. Therefore, a key question to be answered is which kind of *model principles* (or mechanism) drives the emergence of the highly skewed product distribution, *as well as* the earlier discussed typical developmental properties, at the level of excellent human performance.

## Toward a generic model of excellent human performance

In this article we will demonstrate a generic model of ability development that generates characteristic properties of excellent human performance. More specifically, we will discuss a model that underlies the emergence of the typical idiosyncratic developmental properties, as well as the highly skewed productivity distributions across the population, at the level of human abilities and excellent performance.

Because excellence typically develops over time (often over more than 10 years; Ericsson et al., 1993, 2007), we propose a dynamic model of growth to account for a performer's ability development (cf. Van Geert, 1991, 1994). Although such models have not yet been applied to excellence development, some authors already hinted toward their value (e.g., Abbott and Collins, 2004; Abbott et al., 2005; Davids and Baker, 2007; Dai and Renzulli, 2008; Phillips et al., 2010; Aguinis and O'Boyle, 2014). In line with the consensus among researchers that excellence is influenced by various (possibly multiplicatively interacting) personal and environmental variables, we will demonstrate a dynamic network model, according to which abilities leading to excellent performance emerge out of the iterative interactions among multiple variables.

### A dynamic network model representation of ability development

The general idea of a dynamic network model is that higher-order properties are emergent phenomena, that is, patterns of order and structure that emerge on the basis of the dynamic interactions between lower-level components (e.g., Watts and Strogatz, 1998; Strogatz, 2001; Newman, 2003; Barabási, 2009). Within the field of psychology, the feasibility of network models has recently been demonstrated by, among others, Borsboom and colleagues, in their attempts to explain the development of mental disorders (e.g., Cramer et al., 2010; Borsboom et al., 2011; Borsboom and Cramer, 2013; Bringmann et al., 2013). These authors defined the components of the networks on the level of symptoms, such as sleep deprivation, irritability, concentration problems, and so forth. They showed that the psychopathological conditions (e.g., generalized anxiety disorder, depressive disorder) and general properties such as comorbidity are emergent phenomena, originating from the causal interactions on the symptom level (see also Wichers, 2014). Furthermore, in the domain of intelligence Van Der Maas et al. (2006) showed how the “*g*-factor” in intelligence emerges as a consequence of dynamic interactions between lower level components of intelligence, such as perceptual, memory, decision, and reasoning processes. In brief, what these previous network model applications have demonstrated is that interesting theoretical and empirical conclusions can be drawn, without having to specify neither the exact nature of each of the components, nor the exact nature of each of the relationships between the components. Hence, when modeling dynamic networks, it is first and foremost important to specify the general network properties, in particular the principles of *dynamic interactions* between components (i.e., variables) over time, from which a variety of more specific applications can be inferred.

The model that we will discuss in this article can be considered a *dynamic network model* of ability development. Here, an individual's ability network consists of one node (i.e., variable) representing the domain-specific ability, and other nodes that positively or negatively affect the ability (and each other). In line with the existing models in the domain of excellence and talent development (e.g., the Differentiated Model of Giftedness and Talent; Gagné, 2004), the nodes might be of an internal or of an external nature, such as domain-specific interest and family support, respectively. Furthermore, connections between the variables can be supportive or competitive, symmetric or asymmetric. For example, if domain-specific persistence positively affects the domain-specific ability and the ability positively affects the persistence, the connection is symmetric and supportive. Connections may also be either direct or indirect. Consider the example of a good math teacher who positively affects a student's math knowledge, which in turn positively affects the student's learning of physics. In this case the relation between the support of the math teacher and the student's physics understanding is indirect. Any variable in the network is directly connected with a relatively small number of other variables and indirectly connected with a considerably greater number of other variables (cf. Watts and Strogatz, 1998).

The network is dynamic in the sense that the values of the nodes (the levels) change, among others as a consequence of the interactions with other nodes, and nodes may appear or disappear over developmental time (cf. Barabási, 2009). The nature and strength of the relationships between the ability component and other supportive or competitive components in the network are assumed to be idiosyncratic and characteristic of a particular person's dynamic network profile (specificity of ability profile and individual differences are characteristic of excellent performance in general, e.g., Achter et al., 1996; Vaeyens et al., 2008; Phillips et al., 2010; Robertson et al., 2010; for a general discussion of the importance of idiosyncratic models, see Molenaar, 2004; Molenaar and Campbell, 2009). Accordingly, the relationships between components in the network can take various forms (cf. Van Geert, 1991, 1994, 2014). This entails that a component, which exerts a negative influence on a particular ability in one person might have a zero-effect or even a positive effect in another person. For instance, having parents that encourage a child to develop a career in sports may negatively affect the person's academic career, because the parents may not challenge the child sufficiently in solving scientific problems and may let the child devote more time to practicing sports. However, in another child's ability network, the investment in doing sports may improve the child's self-regulation skills, which might have a supportive effect on scientific achievements (Jonker et al., 2011). Related to this, there may be many different developmental trajectories that lead to similar ability levels (Simonton, 1999a, 2000, 2001; Abbott and Collins, 2004; Abbott et al., 2005; Davids and Baker, 2007; Vaeyens et al., 2008; Phillips et al., 2010; Elferink-Gemser et al., 2011; Gulbin et al., 2013).

**Figure 4:**
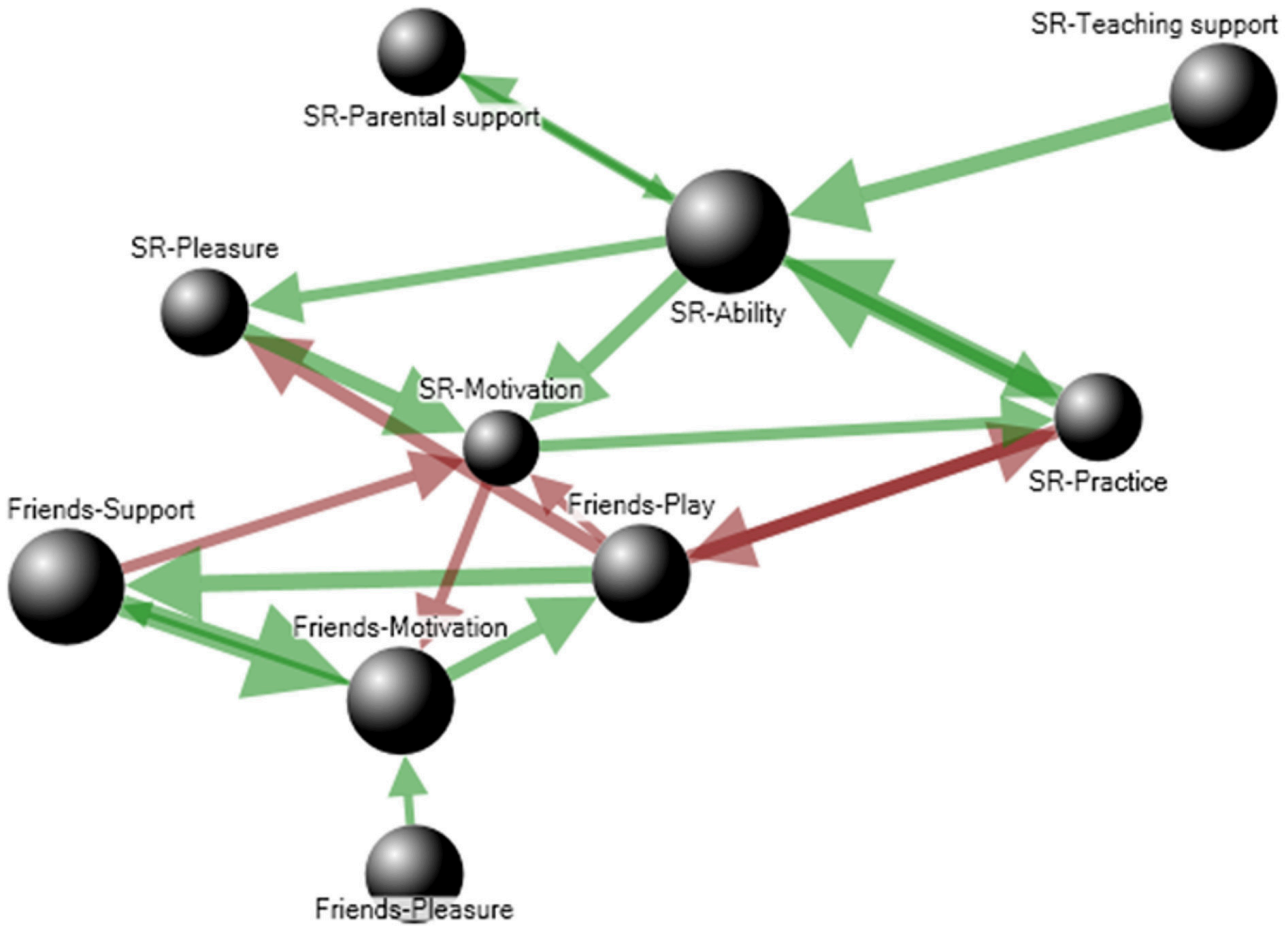
**Graphical representation of an ability network**. Green arrows represent uni- or bidirectional positive influences from one node on another, red arrows represent negative influences.

To provide a simple example of an individual's ability network dynamics, imagine that a particular child has a keen interest in elementary science (e.g., studying insects with a magnifying glass, building marble tracks, etc.). The parents recognize the child's science reasoning ability and stimulate this. To the extent that their child's ability improves, the parents will tend to buy more children's books about science, take the child to museums, and so forth. In addition, the child in this example is strongly intrinsically motivated to work on science and physics problems by means of scientific reasoning. Furthermore, the child also experiences considerable pleasure with solving science problems. This pleasure increases as the knowledge and insights in science increase. In turn, the pleasure further increases the child's motivation for working on science problems and exploration. Then, at secondary school the child meets new friends who like to hang out after school. After having joined the friends for the first time, the child obtains more support from the friends, for example in the form of increasing popularity in the group. In this particular network, hanging out with friends competes with scientific ability development, for instance through a competition for available time (after school) or through a competition between motivation for hanging out with friends and motivation for science learning. If we now take a look at this individual's scientific ability network, the interconnected variables can be displayed in the form of a directed graph consisting of nodes and arrows (Figure 4). Each node corresponds with one component in the child's ability network, and the color of the arrow represents a level of support (green) or competition (red) between two components.

Having explained how dynamic networks of ability development—which take different forms for different individuals—can be visualized, we shall briefly elaborate on the general mathematical principles underlying the dynamic network model, which form the basis for the simulations that we will run to provide a test of the validity of the model.

### Mathematical principles of the dynamic network model

The various nodes and their connections are expressed in the form of equations. All nodes—including the domain-specific ability node—are specified in terms of a growth equation, defining that growth of the variable in question depends on: (1) the level (*L*) already attained, (2) available resources that remain relatively constant during development (*K*) such as genetic endowment (Van Der Maas et al., 2006; Van Geert, 2014), (3) resources that vary on the time scale of ability development (*V*), examples of which could be parental, teaching or coaching support, practice, and tenacity, (4) the degree in which a variable profits from the constant resources (*r*), (5) the positive, negative, or zero weights of the connections (*s*) with the variable resources, and (6) a general limiting factor (*C*) (Van Geert, 1991, 1994). The *C*-parameter is the ultimate carrying capacity, which specifies the ultimate or physical limits of growth of a particular variable. Within the ability network, the variable resources (*V*) are potentially co-dependent on any arbitrary subset of other network variables (including the domain specific ability variable influenced by these variable resources).

The model can be said to implicitly follow a gene × environment approach, that is, the model specifies a multiplicative relationship between the ability-specific *K*-parameter (genetic endowment), and the influence of the support-competition factors in terms of the variable resources (*v*). In light of existing models, the ability-specific *K*-parameter may thus be considered as a kind of “giftedness” parameter. This parameter dynamically moderates the effects of the variable resources, the multiplicative dynamics of which may ultimately lead to expressions of excellent performance (cf. Simonton, 1999a; Gagné, 2004). Note, however, that a portion of the genetic factors may also be present in the variable resources if one proceeds from an epigenetic model in which gen-expressions are influenced by experiences and development (e.g., Simonton, 1999a; Moore, 2015), but this discussion is beyond the scope of the current article.

Furthermore, the network model is a so-called neutral generative model, which means that the weights of the connections *s* are variable and randomly distributed, with an average of zero. Here, we base our reasoning on a simple network model, which does not make specific assumptions about the nature of the connections. The reason for this is that, from a model building perspective, it is important to study the simplest model possible that can generate typical properties of the phenomenon of interest (i.e., excellent human performance). Altogether, the dynamic network model can be mathematically defined as a set of (sparsely coupled) logistic growth equations, each of which represents the growth of a single variable (*A, B, C*, and so forth), and one of which is the domain-specific ability. The number of variables to which a particular variable is connected, is represented by *i, j*, etc:

(4) $$\begin{array}{l} \left\{\begin{array}{l} \frac{\Delta L_{A}}{\Delta t}=\left(r_{L_{A}}L_{A}\left(1-\frac{L_{A}}{K_{L_{A}}}\right)+\sum_{v=1}^{v=i}s_{v}L_{A}V_{v}\right)\left(1-\frac{L_{A}}{C_{A}}\right)\\ \begin{array}{l} \frac{\Delta L_{B}}{\Delta t}=\left(r_{L_{B}}L_{B}\left(1-\frac{L_{B}}{K_{L_{B}}}\right)+\sum_{v=1}^{v=j}s_{v}L_{B}V_{v}\right)\left(1-\frac{L_{B}}{C_{B}}\right)\\ \ldots \\ \ldots \\ \ldots \end{array} \end{array}\right\} \end{array}$$

Taking the illustration of the child in the previous section, each (changing) node corresponds with one equation, representing that variable's growth. All arrows represent the strength and direction (positive or negative) of an influence from one particular variable onto another one. In order to use the network model to simulate lifespan trajectories of ability development, we need to constrain the values of the parameters in the equations describing the changeable values of the network components, which we shall do in the next sub-section.

#### Default model parameter settings

For simplicity, the initial parameter values that we will use for our first simulations are drawn from symmetric distributions. The actual parameter values have no intrinsic or absolute meaning, but are chosen in such a way that the total set of parameters allows us to run feasible simulations of ability development, given the chosen number of simulation steps (e.g., Van Geert, 1994, 2003; Van Der Maas et al., 2006). This means that the values of the parameters have their meaning in relation to each other, rather than some absolute standard (Van Geert, 2014). Recall that, in this article, our aim is to propose general, underlying *model principles* from which excellence emerges, which means that we are not (yet) focusing on the more or less exact *values* of the components as they may exist in domain-specific populations of excellent performers.

Table 1 displays the default distributions from which the parameter values were drawn for each variable in the network, embedded in Equation (4). The standard size that we shall explore in this article consists of 10 nodes. In this network, the number of ability affecting changeable variables thus ranges between 0 (highly improbable) and 10 (also highly improbable). Obviously, in practice many more components may affect the ability, but in terms of the number of components that exert a more or less lasting and significant contribution, 10 seems like a reasonable, defendable maximum that covers virtually all possible cases of excellence development. The in-degree of the nodes, that is to say the number of nodes or variables connecting to any particular variable, is random. With a probability that any two nodes are actually connected set to 0.25 (see Table 1), a 10-node network corresponds with an average in-degree of 2.25 with a standard deviation of 1.3. That is, on average every performance variable is dynamically related to 2–3 variables, but this number may vary between zero and five. The probability value of 0.25 was chosen based on the idea that this value is sufficient to cover all realistic cases of influences on the ability. Note, in addition, that the number of *indirect* links between variables in the network can be exponentially great (cf. Watts and Strogatz, 1998).

**Table 1:** **Default parameter values used for the dynamic model simulations**.

**Parameter**	**Average**	**Standard deviation**
*r* (resource consumption rate)	0.05	0.01
Connection strength with other variables	0	0.02
*K* (stable resources)	1.00	0.15
Connection probability with other variables	0.25	–
	Minimum	Maximum
*L* (initial level)	0	0.05
Time of initial emergence of a variable	1.00	350.00
*C* (carrying capacity)	10.00	25.00

Each iteration or step of the model corresponds with a certain time step or a certain amount of time. Since every model simulation consists of 500 steps, the duration corresponding with a step length of a week is a bit less than 10 years, whereas a step length of about 5 weeks corresponds with a duration of 50 years. A step length of 5 weeks can, for instance, be chosen for the domain of arts or science, in which ability growth and maintenance may cover around 50 years.

Finally, in line with the definition of ability that we provided earlier—focusing on the coordination of different components that are required to demonstrate a particular level of performance—, we identified one node (node 3) as the target variable whose change reflects the ability-development over time. A first step in the validation of the network model, which we shall conduct in this article, is to determine whether the patterns of ability growth that are generated by the model are indeed corroborated by the literature.

## Simulations of developmental properties

In order to check the validity of the dynamic network model, in this section we will examine the model predictions with respect to the development of excellence. More specifically, we will retrieve the properties of (individual) ability development according to earlier literature, and show that all these properties emerge from the dynamic network model simulations.

### Ability development simulations

**Figure 5:**
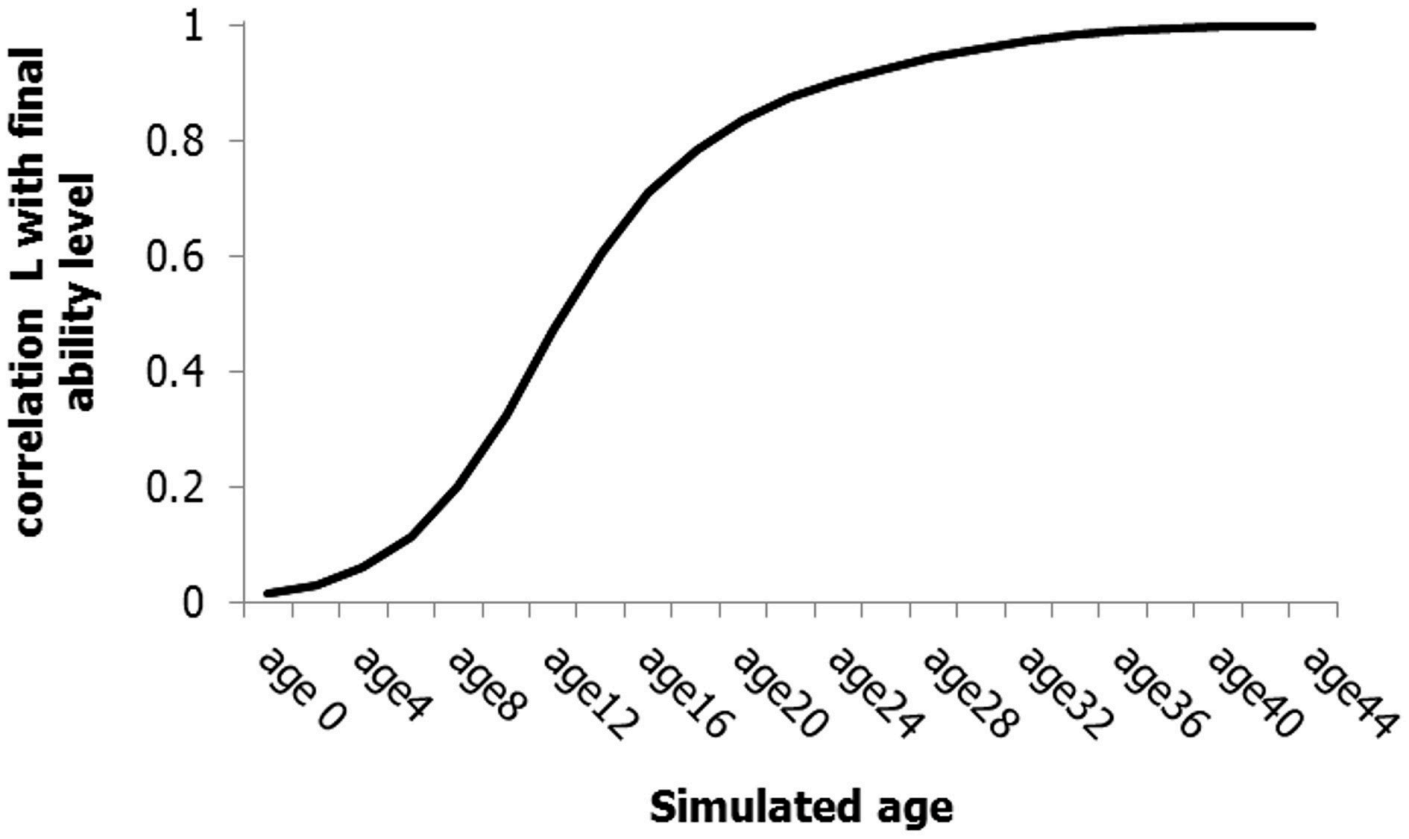
**Correlations between the final ability level and earlier levels based on a set of 1000 simulated life spans**. For simplicity, the simulation steps are expressed in terms of age.

The first property concerns the lack of early indicators or predictors of later excellence (e.g., Howe et al., 1998; Simonton, 1999a, 2001; Abbott and Collins, 2004; Abbott et al., 2005; Davids and Baker, 2007; Vaeyens et al., 2008; Phillips et al., 2010). After simulating 1000 individual cases, we calculated the correlation coefficients (Pearson *r*) between the end-level of the ability-node and the level of this node at earlier simulation steps (see Figure 5). The simulation results show that the correlation with the final level is virtually zero at the beginning, which supports the observation that early indicators of later excellence are often lacking (e.g., Howe et al., 1998; Simonton, 1999a, 2001; Abbott and Collins, 2004; Phillips et al., 2010). Moreover, the correlation reaches a value near 0.5 at the simulated age of 12 years. Qualitatively speaking, this increasing correlation is in line with many studies that show at least moderate to good predictability of later performance around adolescence (Schofield and Hotulainen, 2004; Howard, 2008; Goldstein and Winner, 2009; and in particular studies by Lubinski and colleagues: Lubinski et al., 1996, 2001, 2006; Shea et al., 2001; Wai et al., 2005; Park et al., 2007).

**Figure 6:**
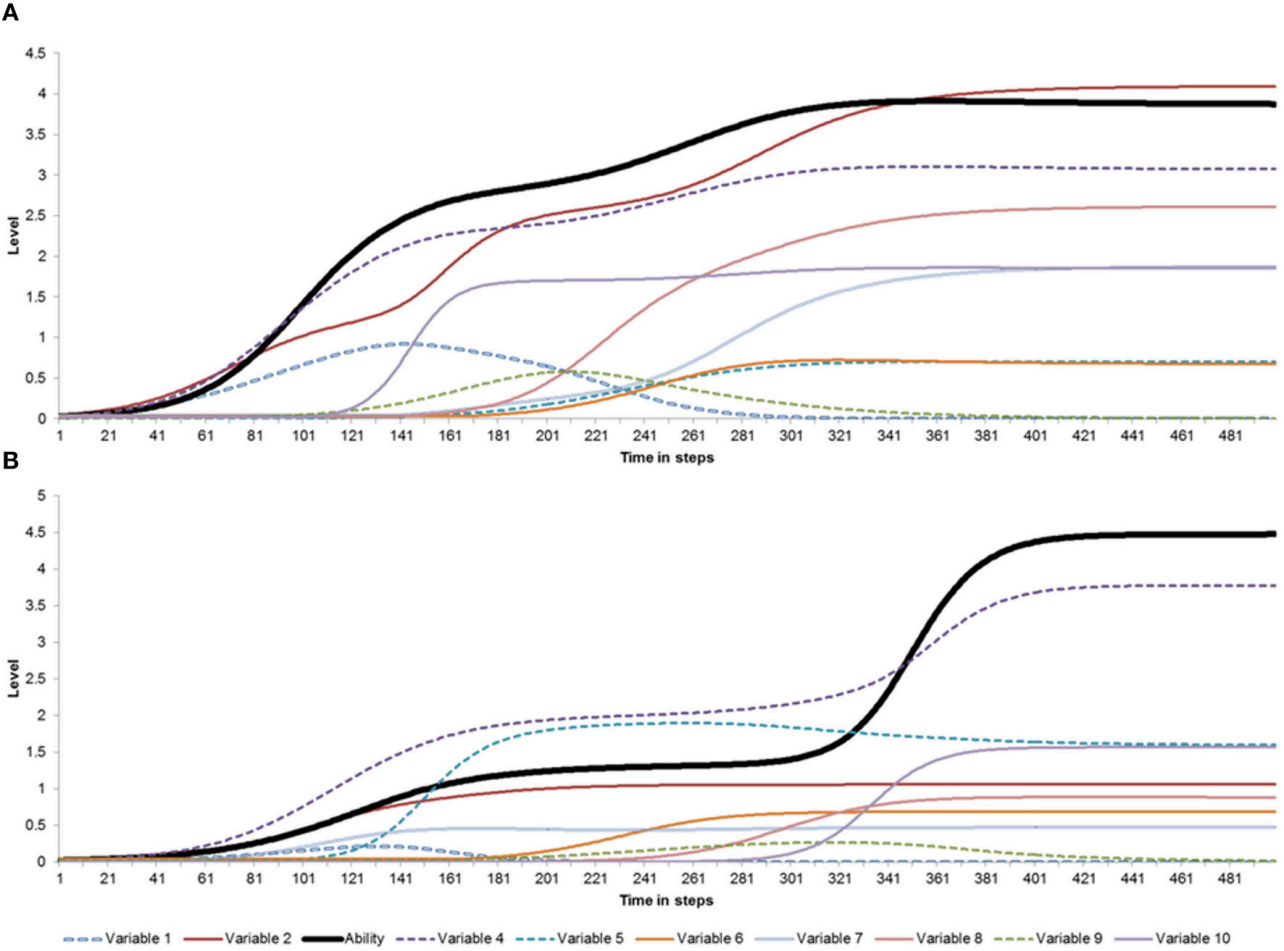
**Simulations of two idiosyncratic networks of individuals who reach high ability levels**. The black solid line represents the ability, whereas the other variables represent the dynamic network components that have supportive, competitive, or neutral relationships with the ability component. For individual **(A)** the initial ability *L* is 0.028; the maximum value of *L* (Max *L*) = 3.91; the resource consumption rate *r* is 0.04; the genetic factor *K* is 1.15; and the total support is 0.07. For individual **(B)** the initial ability *L* is 0.02; the maximum value of *L* (Max *L*) is 4.48; the resource consumption rate *r* is 0.03; the genetic factor *K* is 1.17; and the total support is 0.04.

Figure 6 provides an illustration of how the model is able to simulate the second and third property of excellent ability development, namely that the same type of ability can emerge at different ages in different persons, and that the same ability can be the product of very different underlying factors. The two graphs represent two individuals that, according to the simulation, reach a high ability level (2.85 and 3.46 standard deviations above the mean simulated population ability-level of 1.26, respectively; see Section Simulation Results). However, Person A displays a clear increase early in development, which stabilizes around step 320, whereas the ability of Person B shows a steep increase in ability-level around step 320. Moreover, the genetic component played a relatively larger role in Person B than in Person A (*K* = 1.17 and 1.15, respectively), whereas the supportive factors played a more prominent role in Person A than in Person B (*V* = 0.07 and 0.04, respectively).

The fourth property is that the level of ability is not necessarily monotonically rising or stable: It can change or even disappear during a person's life span. These properties can also be found in Figure 6. For both person A and B there is an overall increase from the start till the end of the ability development. However, in both cases the increase cannot be characterized as a linear growth curve. In addition, the simulations revealed that new variables can emerge at various moments during development (see for instance variable 10 in Person A, which emerges relatively late). These new variables may establish a variety of connections with the already existing variables (cf. the discussion of early vs. late bloomers, e.g., Simonton, 1999a, 2001; Vaeyens et al., 2008). Therefore, the emergence of a new variable may sometimes have a cascading effect on the network, which can lead to relatively sudden changes. An example could be the abrupt increase in ability of Person A when variable 10 emerged. The emergence of a new variable may correspond to, for example, a coach who enters the individual's life, and greatly supports the individual's commitment and ability while working on a sports career. In case a positive feedback loop already existed between commitment and ability, the effect of the new variable (e.g., coaching support) can be greatly amplified.

**Figure 7:**
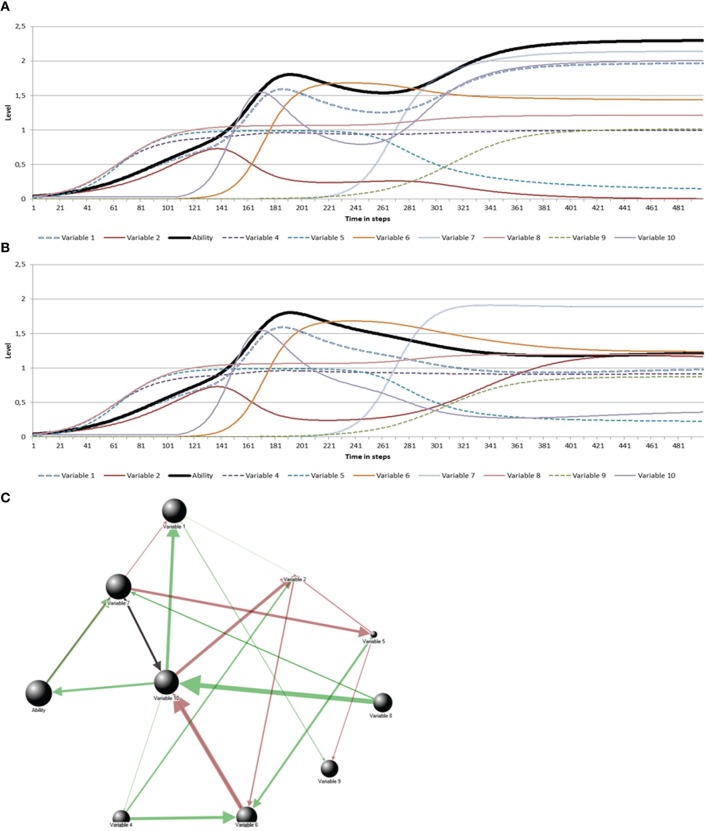
**Two lifespan trajectories**. The difference between the trajectories in Graphs **(A,B)** is caused by the sign (positive or negative, respectively) of the relationship between variable 7 and variable 10 (black arrow in **C**). The parameter values corresponding to the simulations are: Initial ability *L* = 0.041; Max *L* = 2.30 **(A)** and 1.80 **(B)**; Rate *r* = 0.04 **(A)** and 0.01 **(B)**; Genetic factor *K* = 0.99; and total support = 0.03.

However, note that the emergence of a new variable may also lead to a decline of an individual's ability, if the new variable exerts a negative influence on the ability variable or on one of its connected variables. An example of such a phenomenon is the emergence of romantic or sexual interests in peers during adolescence, which may negatively affect the high athletic abilities the adolescent had developed (cf. Csikszentmihalyi et al., 1993). The negative effect of the newly introduced variable need not be direct, since any effects on an important supportive variable will diffuse through the system of network connections, and as a result of this become amplified or eventually damped. Figure 7 illustrates how a difference in the sign (from positive to negative) of one connection—not necessarily that with the greatest strength and not involving the ability variable—changes the pattern of ability development. However, the majority of variables in this specific network (e.g., variables 6 and 7) are relatively insensitive to differences in a single relationship between the nodes of the network, that is, the developments of these variables are not affected.

### Predictions regarding genetic influences on excellence

**Figure 8:**
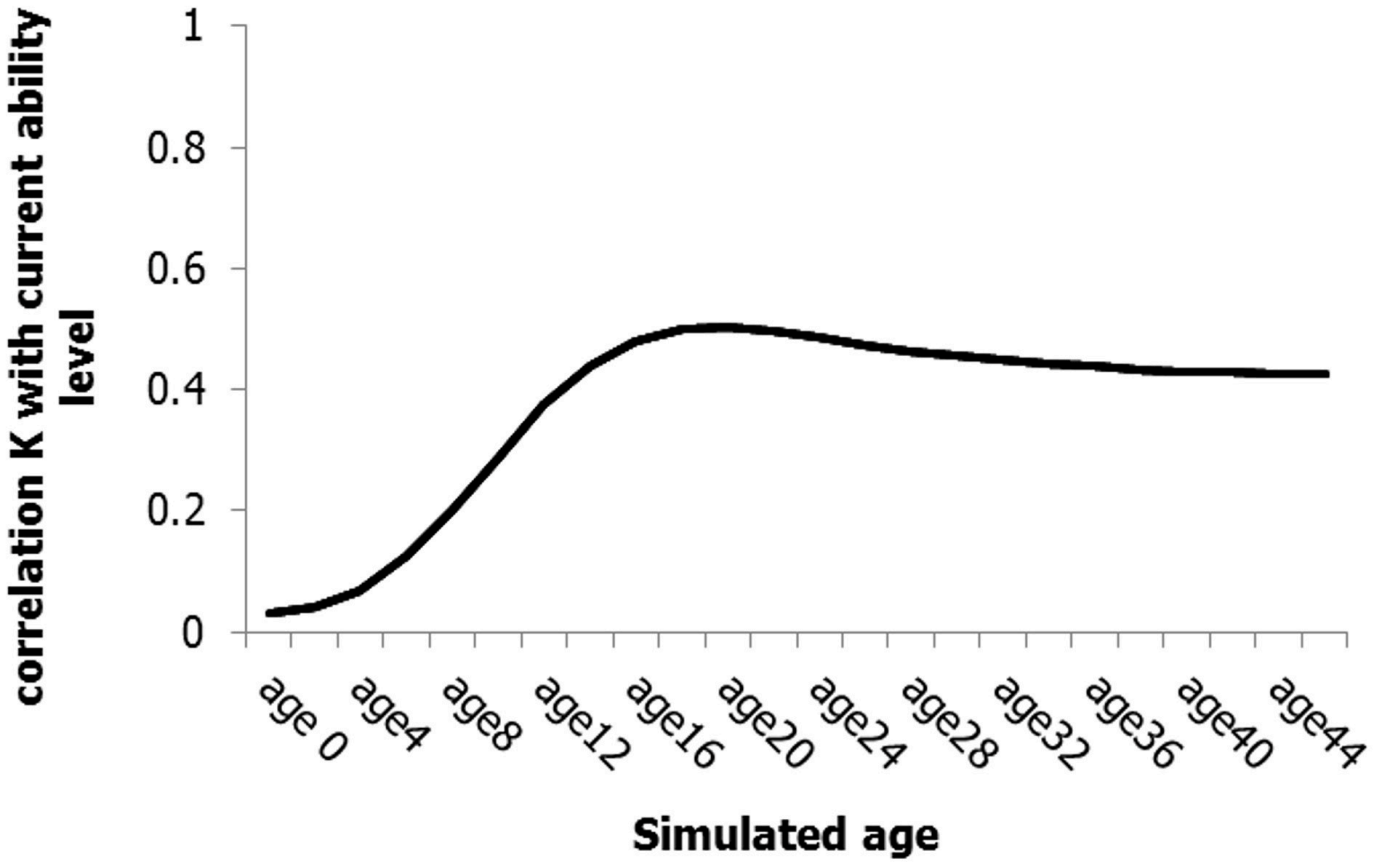
**Correlations between the ***K***-parameter and the ability level at different simulated age-steps**.

As noted previously, the dynamic network model implicitly follows a gene × environment approach, by specifying a multiplicative relationship in the extended logistic growth equation between the ability-specific genetic endowment (*K*), and the influence of the support-competition factors in terms of the variable resources (*V*). Given its prominent place in the literature on excellence, we consider the relationship between genetic endowment and ability as one of the qualitative aspects of ability development that the model should be able to shed light on. To test the relationship between genetic constraints and ability, we determined the correlation (Pearson *r*) between the (genetic) *K*-parameter of the ability-component and the actual ability-level during development. Simulating 1000 individual ability networks, the model predicts that the correlation between the genetic constraint (*K*) and the levels of the ability variable generally increases with age (see Figure 8), a prediction corroborated by previous research (e.g., Bergen et al., 2007; Davis et al., 2009; Haworth et al., 2010). More specifically, in our simulation the correlation first increases to about 0.5, and then falls back to stabilize around 0.4. This specific model prediction is, at least qualitatively, in line with empirical findings on heritability. For instance, a recent extensive twin study on science performance found a drop in heritability from 64% heritability around the age of 9 to 47% around the age of 12 (Haworth et al., 2009). According to these authors, the drop is caused by the increase in environmental effects on science performance after the age of 9. This finding and the suggested explanation are consistent with the network model, in which potentially competitive or supportive variables are stochastically added to the network as age increases—the time of initial emergence of a variable varies between 1 and 350, see Table 1—, thus reducing the relative importance of the genetic component. More generally, while the genetic contribution to developing excellence is currently under debate (e.g., Ericsson, 2013, 2014; Gagné, 2013; Ackerman, 2014; Hambrick et al., 2014; Plomin et al., 2014) our simulation results provide an interesting dynamic perspective on this issue.

### Conclusion simulations of developmental properties

A relatively simple dynamic network, consisting of 10 sparsely connected nodes, generates patterns of ability development that are in accordance with the proposed developmental properties of excellent performance development: (a) Early indicators of ultimate excellent abilities are often lacking, (b) similar ability levels can develop at different ages, (c) the underlying constituents of the ability can change during the individual's life span, and (d) the ability-development can take a variety of forms (e.g., gradual, S-shaped, stepwise, abrupt, e.g., Simonton, 1999a, 2000, 2001; Abbott and Collins, 2004; Abbott et al., 2005; Vaeyens et al., 2008; Phillips et al., 2010; Gulbin et al., 2013). In addition, the network model generates plausible simulation results with regard to the role of genetic endowment. Taken together, these results provide support for the claim that the development of abilities that ultimately lead to excellence is based on a dynamic network model, which is typically interaction-dominant (as opposed to the majority of existing, typical component-dominant models). However, the model should be further validated based on quantitative data. Therefore, in the next section we shall test whether the dynamic network model can predict the extremely right-skewed distribution of products among domain-specific populations, which seems a robust and characteristic property of excellent human performance (e.g., Lotka, 1926; Huber, 2000; Huber and Wagner-Dobler, 2001; Sutter and Kocher, 2001; O'Boyle and Aguinis, 2012; Aguinis and O'Boyle, 2014). We shall also test whether the dynamic network model predictions come closer to the empirical product distributions than do alternative null hypothesis model predictions.

## Simulations of the distributions of excellent performance

The second step in the validation of the dynamic network model, is to examine whether the predictions of the distributions of products across domain specific populations correspond to the empirical data (see Sections Product Distributions in Soccer and Product Distributions across Domains). The productivity of actual performers does not only depend on their abilities, but also on a myriad of accidental factors that occur with highly variable probabilities (e.g., Simonton, 2003; Gladwell, 2008; Elferink-Gemser et al., 2011). For instance, in the domain of science, having a paper published in a high-impact journal may depend on the unexpectedly striking results the researcher found or on getting just the “right” reviewers (Simonton, 2003). In order to predict the productivity of an individual, and the productivity distribution across a population of excellent performers, we therefore not only need a model of the underlying abilities, but we also need a model of how abilities lead to the products, thereby taking into account the inherently stochastic nature of performance productivity.

Earlier literature has already suggested several product models that aim to predict the generation of domain-specific products. These product models link a product probability to a particular ability level during some time interval *t*. They are, however, not linked to a model of *ability growth*, like the one we discussed in preceding sections. In other words, existing product models predict product generation, but do not tell where the abilities to generate products come from. To further test the validity of the dynamic network model, we will combine the growth of the ability variable with existing product models. Before discussing the model simulations of productivity, we will first give a short overview of existing product models that have been supported by empirical data in previous literature.

### Existing models to predict the generation of products

The product model discussed most in the literature is the Poisson model, which may occur in various forms. The simplest Poisson model, in particular introduced by Huber (e.g., Huber, 2000; Huber and Wagner-Dobler, 2001), states that the probability (*p*) that a particular product such as a goal in a soccer match or a scientific paper will occur during a fixed time interval *t*, is the mathematical product of a domain-specific Poisson parameter φ and the individual's current level of the underlying ability, L:

(5) $$\begin{array}{l} p\left({\text{P}}_{t}\right)=\varphi {\text{L}}_{t}. \end{array}$$

For different performance domains, φ is typically a very small value, which is in accordance with the Poisson nature of the process. The model has also been presented as the blind-variation-and-selective-retention model (BVSR model, Campbell, 1960; Simonton, 1999b), or as Simonton's equal odds baseline, which explains exceptional performance as a constant probability of success depending on the level of the underlying ability. The simple Poisson model has been empirically corroborated in a variety of fields, in particular scientific and musical creativity (e.g., Dennis, 1954, 1956; Simonton, 1984, 1991, 1997, 2003).

A second product model is based on the assumption that total productivity is a function of an ability component (L), and a tenacity (domain-specific persistence, commitment) component M (see Huber, 2000, for career longevity in sports, science and technological creativity; Petersen et al., 2008, 2011, in the field of sports). Hence, the probability that a product is generated depends on the Poisson parameter and the product of the ability- and tenacity-level.

(6) $$\begin{array}{l} p\left({\text{P}}_{t}\right)=\varphi \text{LM} \end{array}$$

Another product model is Simonton's model of creative potential, which applies to creative talents such as the arts, sciences or technological inventions (Simonton, 1984, 1997). The model states that a particular creative ability corresponds to a creative potential, that is, the potential to generate *n* products. Given a particular ideation rate (rate of idea production), the number of products generated per unit time is a function of the potential (L) and of a depletion factor, namely the number of products already actualized (L′_*t*_). Simonton's original model of three coupled differential equations can be simplified as follows:

(7) $$\begin{array}{l} p\left({\text{P}}_{t}\right)=\varphi L\left(1-\frac{{{\text{L}}^{\text{′}}}_{t}}{\text{L}}\right) \end{array}$$

The final product model, the so-called Matthew model, is based on the success-breeds-success principle. In the educational sciences, the Matthew effect may refer to situations where a child with an observable talent for math or arithmetic, for instance, is likely to attract the interest of parents or teachers who tend to invest special effort helping the child develop this talent (Bloom, 1985). In sociology, the model has been applied to exceptional scientific productivity (e.g., Merton, 1968; DiPrete and Eirich, 2006). Merton (1968), for instance, proposed that the scientific community favors those scientists who have been most successful in the past by providing them with additional resources and attention, thereby further stimulating their productivity (for recent quantitative evidence for the Matthew effect in scientific careers, see Petersen et al., 2010). Accordingly, typical of the Matthew model is that the probability of a particular output increases as a function of the number of outputs already produced. Hence, if S_*t*_ is the number of products already produced at time *t*, the Matthew product model becomes equal to

(8) $$\begin{array}{l} p\left({\text{P}}_{t}\right)=\varphi \left(1+\gamma {\text{S}}_{t}\right){\text{L}}_{t} \end{array}$$

(the γ parameter is a scaling factor, moderating the effect of the number of products).

### Predictions regarding product distributions

To predict the number of domain-specific products that is released over an individual's career, we can embed the product models in the dynamic network model, by coupling a particular product model per unit time to the long term change in the ability variable. By doing so we can simulate not only the temporal trajectory of the ability, but also the temporal trajectory of the productivity based on that ability, including the total life time productivity of a simulated individual.

In order to test the validity of the predictions that the network model will make about the distribution of the number of products of a certain kind, we need to compare the network model with a null hypothesis model. The null hypothesis model is based on the standard statistical assumption that abilities are normally distributed across the population, and result from additive effects of major performance-related components, such as motivation, coaching, and practice. Therefore, we reduced the connection strength with other variables to 0, and treated the *K*-parameter of the ability variable as the parameter including *all* resources to develop excellence:

(9) $$\begin{array}{l} \frac{\Delta L}{\Delta t}=rL\left(1-\frac{L}{K}\right). \end{array}$$

#### Model settings

In line with our minimalistic approach—to check whether a network model with the simplest, least specific assumptions—already has sufficient explanatory power, in this section we shall confine ourselves to adding the simplest possible productivity model [the simple Poisson model, see Equation (5)] to the network model. We will test whether this model suffices to predict the major properties of excellent productivity, in particular the strongly right-skewed distribution. Because, in accordance with the empirical distributions, the majority of (excellent) performers in a specific domain has one product (e.g., Lotka, 1926; Huber, 2000; Sutter and Kocher, 2001; O'Boyle and Aguinis, 2012), the probability that a product is released during each time step is chosen in such a way that the mode of productivity during an entire life span is 1. The Poisson parameter that corresponds with this lifetime average is 0.002, since the simulation length was set at 500 steps. Each simulation round of the model represents a life cycle of an individual, randomly drawn from the possible population of individuals who have the ability to produce at least one product of interest (e.g., a scientific article, a patented invention, a goal in an international soccer match). Thus, the model simulates a population in which a particular domain-specific ability—playing soccer, doing research, playing violin, etc.—actually develops.

#### Simulation results

**Figure 9:**
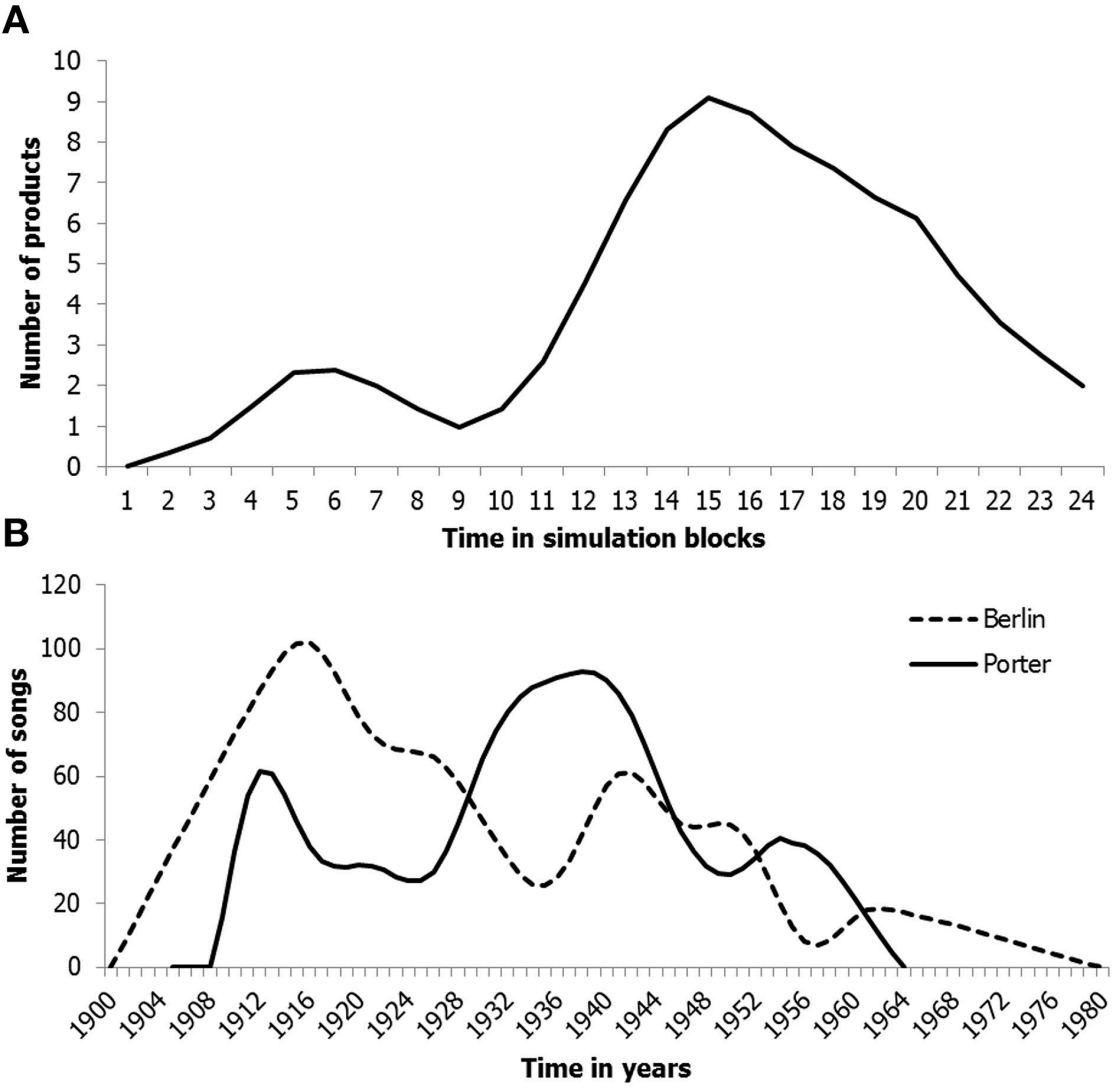
**Illustration of a single individual's simulated productivity across the life span (A) and the life span productivity of the songwriters Irving Berlin and Cole Porter (B)**. In **Graph (A)** the time axis corresponds to time blocks, i.e., 24 periods of 20 time steps.

To start with, simulating the productivity over the lifespans of single individuals reveals nonlinear, often quite variable trajectories (see Figure 9A for an illustrative example). Although reports on productivity over the lifespan are scarce, data on the lifespan productivity of two prolific songwriters, Irving Berlin and Cole Porter, provide qualitative support—also demonstrating peaks and valleys across the life-span—for the predictions of the network model (Hass and Weisberg, 2009; Hass et al., 2010; see Figure 9B).

However, the primary goal of the productivity simulations is to check if the predicted number of products across a *simulated population*—based on the simplest production model—resembles the highly skewed distributions according to the large body of empirical data discussed earlier. Furthermore, we shall test whether the predictions of the network model are more accurate than those of the null hypothesis model. More specifically, we will examine whether the network model predicts a right-skewed distribution with the correct empirical properties, namely a distribution described by a stretched exponential or power law, and that the null-hypothesis model does not do so. To provide a reliable comparison between the network model results and the null-hypothesis results, we first augmented the average level of the *K* component of the null-hypothesis model from 1 to 1.26, which is equal to the average ability level resulting from 100,000 simulations of the network model with the default parameter settings.

**Figure 10:**
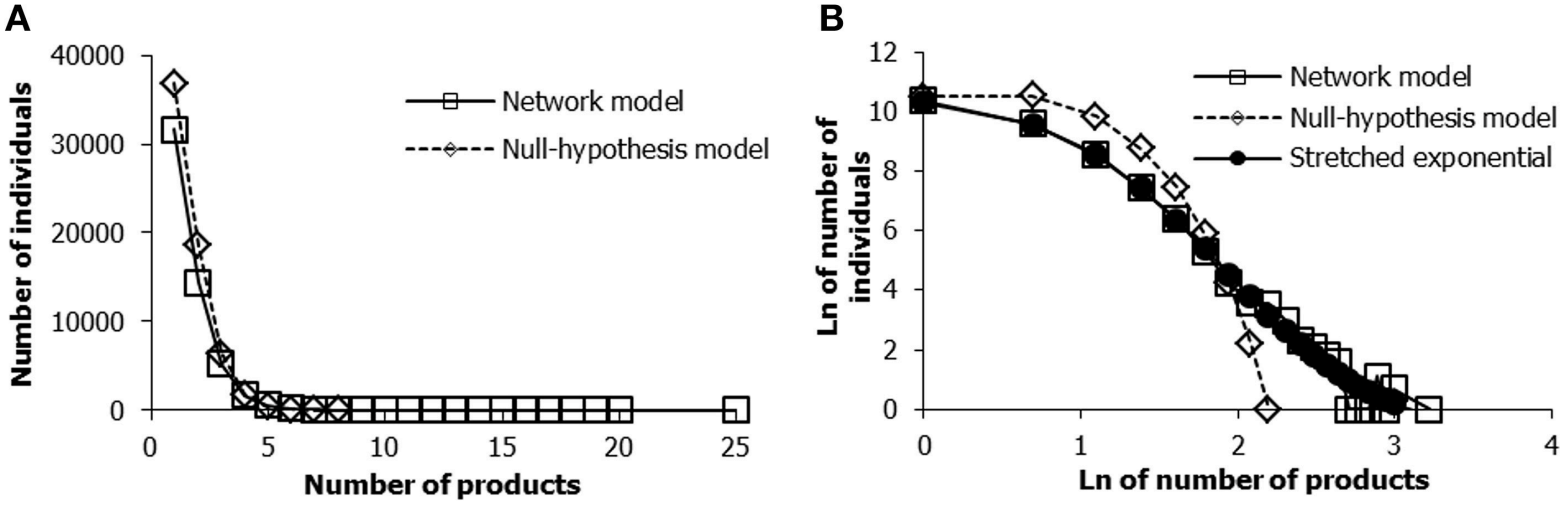
**Simulated product distributions of the network model and the null-hypothesis model**. Graph **(A)** displays the raw frequencies. In Graph **(B)** the data are plotted according to the natural logarithmic scales.

Results of the simulation show that, first, both the network and the null hypothesis model generate right-skewed product distributions (Figure 10). However, consistent with the data described earlier, the right tail of the product distribution generated by the network model is considerably longer than that of the null hypothesis model. In the simulation this amounts to a maximum of 25 in the network model vs. a maximum of 8 products in the null hypothesis model (see Figure 10A). The right part of the distribution based on the network model thus shows the characteristic right tail corresponding to the truly exceptional cases, as revealed by the empirical data described earlier. Furthermore, the log-log distribution of the products and frequency as generated by the network model is similar to a stretched exponential distribution, which is not the case for the null-hypothesis model (Figure 10B). The distribution as generated by the network model is typical of virtually all the data on product distributions discussed previously (see Sections Product Distributions in Soccer and Product Distributions across Domains).

#### Testing the null hypothesis

Before we can reject the null-hypothesis model in favor of the network model, however, we must ask if it is possible to alter the parameter values in such a way that also the null hypothesis model can produce the empirically expected product distributions (a possibility that would greatly reduce the validity of the network model). The first important parameter we should consider is the *K*-parameter. As noted earlier, the *K*-parameter in the null-hypothesis model represents the *sum* of genetic resources and other (environmental) resources. Can its standard deviation be increased in such a way that also the null hypothesis model generates the product distributions in accordance with the empirical data? Another important parameter is the Poisson parameter, which determines the probability of generating a product per unit time. In order for the null hypothesis model to generate a *maximum* number of products comparable to that of the network model, we had to set a seven-times bigger Poisson parameter, but also an *average* value of the *K*-parameter that is 25% higher, and a standard deviation of the *K*-parameter that is twice as big. However, with such parameter values, the *average* number of products in the population is about 10 times bigger than generated by the network model, with a distribution that is almost symmetrical. Such characteristics are in complete contradiction with the properties of the empirical skewed distributions of performance products (e.g., Lotka, 1926; Huber, 2000; Huber and Wagner-Dobler, 2001; Sutter and Kocher, 2001; O'Boyle and Aguinis, 2012; Aguinis and O'Boyle, 2014).

In sum, our proposed network model succeeds in producing the major properties of the available productivity data, namely that they are highly right skewed and that the distribution can be described by a stretched exponential- or power law. The null hypothesis model also produces a skewed distribution of the products, but fails to produce the orders of magnitude that are so characteristic of the empirical distributions. Apart from these valid general predictions, as a final step in this article it is useful to examine how the network model may fit within specific performance domains. To obtain a domain-specific fit, the settings of the standard parameters of the network model, as well as of the product model, can be varied as we will briefly demonstrate in the next section.

### Different parameter settings to predict product distributions in specific domains

**Figure 11:**
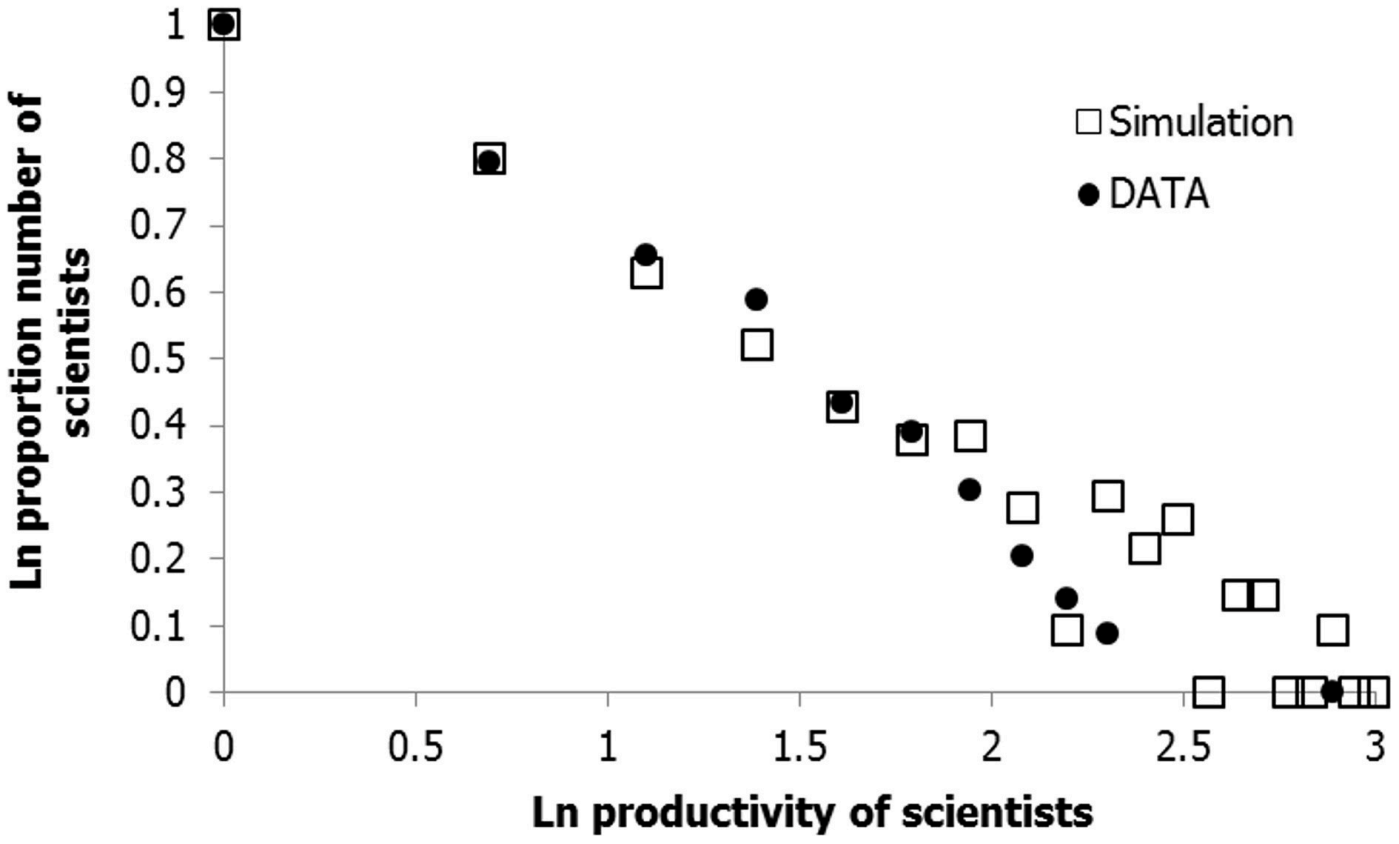
**Log-log plot of the number of articles published in high ranked economics journals against proportional number of authors**.

Figure 11 demonstrates that the model-parameter settings can also be fine-tuned to fit with the domain of scientific performance, based on the empirical data of Sutter and Kocher (2001). More specifically, the model accurately predicts the productivity distribution of economics scientists if the default parameter settings (Table 1) are adapted in such a way that the variable support contribution is augmented relative to the (genetic) *K*-parameter, which, in all likelihood, is quite characteristic for the domain of science (e.g., Krebs, 1961; Berry, 1981; Beaver, 2001). We simulated the distribution of productivity (number of articles) of randomly selected patches of 3637 cases (the total number of authors in the study of Sutter and Kocher, 2001), taken from a universe of 10,000 simulated cases. The *K*-parameter in the network model was reduced from 1.00 to to 0.30 (*SD* = 0.10), whereas the range of the variable support contribution was extended (*SD* = 0.10 rather than 0.02). The product model we used is based on the simple Poisson model (Poisson parameter is a random number between 0.0015 and 0.0025). In addition to the comparable distributional properties, the average and maximal number of publications based on simulation (1.62 and 20, respectively) were close to the average and maximum based on the empirical data (1.44 and 18.00, respectively).

When fine-tuning the network parameters and/or product model to other domains, we also found simulation results in line with the literature, which we shall briefly mention. First, according to the second product model, domain-specific productivity is a function of the domain-specific ability (L) and a tenacity factor (M) (Huber, 2000; Huber and Wagner-Dobler, 2001). The tenacity factor is represented by an arbitrarily chosen variable in the network model, in this case the fourth variable. A domain in which tenacity is considered a major determinant of success, is sports (e.g., Abbott and Collins, 2004; Abbott et al., 2005; Van Yperen, 2009). Based on the ability × tenacity product model (Equation 6), and with network parameter settings adapted to the sports domain, accurate predictions of productivity distributions in tennis and soccer are generated (Den Hartigh, 2015).

Simulations based on Simonton's creative potential model (Equation 7) predict a domain-specific peak in productivity, which is corroborated by empirical data (e.g., Simonton, 1984, 1997). In the network model the place of the peak is determined by the ability level and by two parameters, the creative potential constant (which is multiplied by the ability level), and the depletion constant (which is multiplied by the current number of products already generated). The number of products generated under this model spans various orders of magnitude, which is consistent with the Simonton's empirical data (e.g., Simonton, 1999a, 2003).

Finally, an example of a product model that, connected to the network model, generates an extremely right-skewed distribution is the Matthew product model (Equation 8). When running simulations based on this product model—for convenience we used the network parameters displayed in Table 1—the number of products is highly stretched (that is, the maximum number of products is many times higher than the maximum number of products without it). Such distributions are, however, relatively more often found with respect to the *impact* of products (which is not the primary focus of the current article), as examined in the study of literary fame, for instance (e.g., Martindale, 1995). These extreme distributions could be explained by the success-breeds-success principle, more specifically that the attention that a writer gets from literary critics is primarily determined by how much (positive) attention he or she has already received from other critics (Verdaasdonk, 1983).

### Conclusion simulations of the distributions of excellent performance

The network model of excellent human performance predicts the highly right-skewed product distributions as observed within various domains, if one of the existing product models is coupled with the network model of ability development (e.g., Huber, 2000; Huber and Wagner-Dobler, 2001; Simonton, 2003). Furthermore, it does so with an evidently higher accuracy than the null hypothesis model we have tested. In addition to this general validation, we have shown that domain-specific product distributions can be predicted with high accuracy when adapting the parameter-values and/or product model according to the domain of interest. We may therefore conclude that the validation of the model based on the properties of ability growth we discussed earlier, and on the existing quantitative data of excellent performance in the form of product distributions, was successful.

## Discussion and conclusion

In this article we endorsed the theoretical view that excellence can be considered as multidimensional, and developing from *idiosyncratic, dynamic* relationships among several variables, forming a network. We also worked from the view that excellent performance can be measured in terms of productivity, especially if this productivity is based on consensual expert assessment, as in science, technology, arts and sports (e.g., Simonton, 2003; O'Boyle and Aguinis, 2012). We proposed a generic dynamic model of ability growth, consisting of connected internal and external variables. The relationships between these nodes were defined as dynamic, competitive or supportive relationships, which are randomly and sparsely assigned. Any specific combination of positively or negatively weighted relationships between the performance variables, rates of resource consumption, carrying capacities, and initial levels in principle represents a possible person in the (simulated) population.

In order to check the validity of our proposed dynamic network model, we started with predictions of the properties regarding the development of (excellent) abilities that are generalizable across a wide variety of achievement domains. We showed that the developmental properties of excellence are revealed by simulations of our dynamic model. These properties are: (a) Early indicators of ultimate excellent abilities are often lacking, (b) similar ability levels can develop at different ages, (c) the underlying constituents of the ability can change during the individual's life span, and (d) the ability-development can take a variety of idiosyncratic forms (e.g., Howe et al., 1998; Simonton, 1999a, 2001; Abbott and Collins, 2004; Abbott et al., 2005; Phillips et al., 2010).

Within the model, ability support is not a matter of an addition of supporting variables, but a dynamic process of mutually supporting and competing variables. The effect of such variables is dynamically moderated by stable resources such as the person's genetic endowment for a particular ability. As to the influence of genetic factors, the model predicts an increase in heritability that is followed by a slight decrease, which has been found within domains such as science performance (Haworth et al., 2009). Hence, it also provides a dynamic perspective on the role of genetic endowment in the development of excellence, a topic that is still hotly debated (e.g., Ericsson, 2013, 2014; Gagné, 2013; Ackerman, 2014; Hambrick et al., 2014; Plomin et al., 2014). As our model predictions suggest, heritability—like other ability-related variables—should not be considered as a separate mechanism to explain excellence, but as a factor whose functional role is dynamically embedded in a network consisting of multiple ability-related variables (cf. the discussion on component vs. interaction-dominant dynamics; Van Orden et al., 2003).

To provide further validation for the dynamic network model, based on distributions of excellent performance data as found in the domain of soccer and as revealed in many earlier studies (e.g., Lotka, 1926; Huber, 2000; Sutter and Kocher, 2001; O'Boyle and Aguinis, 2012), we combined the model with one of the existing product models. Products were generated as stochastic outcomes, at each time step, of the ability level and a Poisson productivity parameter. The product distributions as generated by our model resembled the strongly skewed empirical product distributions very well, which clearly contrasted with the null hypothesis model-predictions. Hence, based on the simulation results of both the developmental properties leading to excellence, and the product distributions across populations of excellent performers, it seems highly likely that excellent performance emerges from idiosyncratic, interaction-dominant dynamic network structures.

### Implications of the model

A major strength of the dynamic network model is that it predicts a considerable number of properties typical of excellent human performance in many achievement domains, such as the idiosyncratic ability trajectories and the strongly skewed product distributions across the population. It does so on the basis of a very general model of ability growth that makes only limited assumptions and that is basically neutral in its parameters. On the other hand, one could also argue that the model's major weakness is that the structure of connections that may occur in networks is so rich that virtually any kind of structure may emerge. Therefore, the complexity of the possible relationships make any such network hard to explain, if by explanation we mean determining the exact contribution of separate components on the level of individuals. However, recent work in statistical modeling of idiosyncratic developmental patterns has made considerable advances (e.g., Molenaar and Nesselroade, 2012). Of particular relevance in this regard is the work on reconstructing idiosyncratic networks of interactions between components, in the form of mental states characteristic of psychopathology such as depression (see Borsboom and Cramer, 2013). Such techniques can be applied to any type of network.

Moreover, it is important to note that the explanatory complexity of idiosyncratic individual networks cannot be reduced by replacing the explanandum (the individual system) by a statistical ensemble consisting of a collection of many such individual systems, characterized by statistical relationships between the distribution of properties across this collection (see Molenaar, 2004; Molenaar and Campbell, 2009). Explaining the time-based evolution of such individual systems must begin with attempts toward understanding the general properties of the underlying dynamics as they apply to the individual, idiosyncratic processes. The dynamic network model attempts to do so, and provides insights into population characteristics, such as the distribution of products, by generating many individual trajectories covering a particular population of individual performers. This way, the dynamic network model aims to make a significant theoretical contribution, keeping in mind the argument that the value of a theoretical model lies in its ability to account for empirical observations across a wide variety of contexts and situations (Pierce and Aguinis, 2013). Accordingly, as an interesting future avenue the model simulations can be expanded to predict empirical distributions of fame, impact, or popularity with respect to excellent performance in a particular domain. More specifically, the dynamic network model could be used to model the number of public performances of artists, but also tokens of impact or fame of a person, such as the number of citations of a scientist, books or articles about a particular writer, etc.

A relevant question from both a theoretical and applied point of view, is whether the (potential) ability to reach excellent performance is “in the individual” and therefore must be discovered at an early age, whether it should be elicited by accumulating much practice, or whether it can also be elicited in other ways. The first possibility is still widely embraced and has dominated the “talent detection” programs in research and practice around the world (Ericsson et al., 1993; Howe et al., 1998; Abbott and Collins, 2004; Abbott et al., 2005; Vaeyens et al., 2008; Phillips et al., 2010). On the other hand, the two latter possibilities are in accordance with the view that excellence develops mainly through (variable) factors that support or stimulate the ability (e.g., Ericsson et al., 1993; for popular accounts of this point of view, see Colvin, 2008; Coyle, 2009). However, the predictions of the network model do not correspond to the idea that one specific factor, such as deliberate practice, should be primarily focused on in order to develop excellence (Ericsson et al., 1993). That is, our network model predicts that there are many possible multiplicative relationships between dynamic variables leading to excellent performance. Taken together, while excellence can be elicited and nurtured, the ability growth and final level depends on genetic endowment factors (the *K* parameter in the mathematical model) and on the many possible combinations of variables that support the ability at issue (for a comparable stance, see Simonton, 2014).

Because the network model assumes that excellence can be “fed” by multiplicative relationships between a variety of components that are individual-specific, we may cast doubt on a policy that emphasizes just one or very few links, and/or that applies a “one-size-fits-all approach” [e.g., when children are exposed to a tough sports practice regimen in a (relatively isolated) gymnastics institute to improve their athletic abilities; see also Van Rens et al., 2015, on the lack of effectiveness of schools focusing on sports talents]. However, future empirical studies and simulation studies should be conducted to explicitly test the consequences of different kinds of network structures with regard to the development of excellence. One concrete possibility is a longitudinal study in which a diary is filled out by children themselves and by the parents at regular occasions, in order to reconstruct the dynamic network of components and relationships typical of a particular child from the group of excellent performers (relative to peers).

In relation to this, the practical implications of the dynamic network model primarily relate to the *structure* of the individual ability networks, more so than the exact *nature* of the components that generally relate to excellence across the population. The challenge from an applied perspective is to establish positive links between ability-related components in individual, idiosyncratic trajectories that potentially lead to excellence. That is, the (possible) interactions between the components should be a primary focus of attention, which means that the probability of establishing positive feedback-loops between various ability-supporting components should be enhanced. Accordingly, instead of focusing on isolated and common components such as hours of practice or intrinsic motivation, a coach, or teacher should be sensitive to the child and its environment (e.g., the enthusiasm, engagement in school) that signal supportive or competing influences for excellence to develop. In other words, the coach or teacher should be adaptive, because he or she is situated in an idiosyncratic and changing network that is typical for a particular athlete, artist, or scientist, and which involves mutual interactions between components.

To conclude, there are many possible ways to kindle a fire, and many kinds of fuel to keep it burning. In this article we considered excellence as a developmental and emergent property, and proposed that excellence should be approached from a dynamic network perspective. Population-based studies and theoretical models of associations between predictor variables and excellent performance variables provide important information, but cannot be used as a substitute for time-serial, individual-based studies and process models of excellence emergence and development. Our dynamic network model provides a framework for studying such individual trajectories, but it also provides an explanation for the population-distributions of excellent performance in science, sports, arts, and beyond.

## Author contributions

PV, HS, MV, and RD conceived the conceptual model; PV and RD conducted the data acquisition and model simulations; and RD, PV, MV, and HS drafted the article. RD, PV, MV, and HS have approved the article to be published and agree to be accountable for all aspects of the conducted work.

### Conflict of interest statement

The authors declare that the research was conducted in the absence of any commercial or financial relationships that could be construed as a potential conflict of interest.
